# Traumatic Lateral Knee Dislocation of a Well-Functioning Total Knee Arthroplasty: A Case of Medial Collateral Ligament Rupture

**DOI:** 10.1016/j.artd.2021.08.012

**Published:** 2021-09-30

**Authors:** Kevin Smidt, Matthew Dubose, Cambize Shahrdar

**Affiliations:** a Department of Orthopaedic Surgery, Louisiana State University Health Sciences Center, Shreveport, LA, USA; b Department of Orthopedic Surgery, Willis-Knighton Health System, Shreveport, LA, USA

**Keywords:** Total knee arthroplasty, Complication, Dislocation, Tibiofemoral, Instability, MCL rupture

## Abstract

Tibiofemoral dislocation can be a devastating complication after total knee arthroplasty. Much of the literature on tibiofemoral dislocations state they result from iatrogenic causes, with a very limited number of case reports on traumatic dislocation. Most of the time, these cases will require surgical revision and increased constraint to treat the inherent instability. In addition, collateral ligament disruption increases the complexity of the treatment algorithm for these patients. We report the case of a lateral tibiofemoral total knee arthroplasty dislocation with associated medial collateral ligament injury treated successfully without surgical interventions.

## Introduction

One of the most dreaded complications of an unconstrained total knee arthroplasty (TKA) is injury to the medial collateral ligament (MCL). MCL disruption causes coronal plane instability that leads to accelerated wear, loosening, and failure of an otherwise well-positioned TKA [1,2]. If this complication occurs intraoperatively, an attempt can be made to repair the MCL if the posterior cruciate ligament (PCL) remains intact, otherwise the surgeon must consider conversion to a constrained prosthesis [2,3]. Increasing prosthetic constraint leads to increased stress at the implant-cement and implant-bone interfaces causing osteolysis, accelerated polyethylene wear, and increased risk of aseptic loosening, all of which can be devastating especially in the young, healthy, and active patient populations [4]. Therefore, the most conservative treatment strategy is preferred in the event of MCL disruption during TKA. Leopold et al. demonstrated successful results with primary repair of the MCL during TKA without the need for a constrained prosthesis when combined with 6 weeks of postoperative bracing [5].

Not all MCL injuries occur intraoperatively, however. The MCL may become incompetent because of implant malpositioning or improper ligament balancing, which likewise poses a difficult problem for the surgeon. It has been shown that collateral ligament reconstruction alone as a subsequent operation for the treatment of the unstable TKA is ineffective [6].

Another infrequently reported mechanism of MCL injury in the setting of a previously well-functioning TKA is tibiofemoral dislocation. The prevalence of this complication after primary TKA is estimated at 0.15%-0.5% [[7], [8], [9], [10], [11]]. The vast majority of these tibiofemoral dislocations are anterior or posterior, whereby the tibial component translates anteriorly or posteriorly to the femoral component. Rouquette et al. performed a systematic review of tibiofemoral dislocations after primary TKA and found that displacement was posterior in 87.3% of cases, anterior in 11.4%, and lateral in 1.3% [7]. Jethanandani et al. reported that 86% of their TKA tibiofemoral dislocation cohort required revision surgery, although the authors emphasized polyethylene damage and ligamentous insufficiency as causes of dislocation rather than traumatic injury [12].

There is a paucity of literature regarding traumatic tibiofemoral dislocation in the setting of a previously well-functioning TKA. One such case report by Wang C.J. and Wang H.E. describes a 71-year-old woman who underwent a posterior cruciate-retaining TKA as she twisted her operative knee while getting up from a chair, causing a posterolateral dislocation on postoperative day seven [13]. She was treated with closed reduction and cast immobilization for 6 weeks and had satisfactory stability and function at her 2-year follow-up [13]. To our knowledge, there is no published literature regarding purely lateral TKA dislocation with a previously well-functioning TKA secondary to trauma, and treatment strategies remain unclear. This case report outlines the course of an active 66-year-old male who sustained a traumatic lateral knee dislocation with associated MCL tear after a previously well-functioning highly congruent anterior-stabilized TKA. The patient was informed that the data concerning the case would be submitted for publication, and he provided consent.

## Case history

A 66-year-old male physician with a past medical history of gastroesophageal reflux disease and hyperlipidemia underwent successful simultaneous bilateral TKA using the Zimmer (Warsaw, IN) Natural Knee II implant system with hybrid fixation at another institution. The femoral components were cruciate retaining (CR), press-fit porous implants, and the tibial components were cemented baseplates. The polyethylene inserts were 9 mm ultracongruent (UC) components. The patellae were not resurfaced. The surgeon who performed the total knee replacements had difficulty with exposure and, therefore, performed bilateral quadriceps snips as well as a controlled release of the patellar tendon off the tibial tubercle to sublux the patella laterally. At the conclusion of the surgery, the patellar tendon was repaired using Arthrex (Naples, FL) Corkscrew FT (fully threaded) suture anchors. The PCL was also removed during the approach.

**Figure 1:**
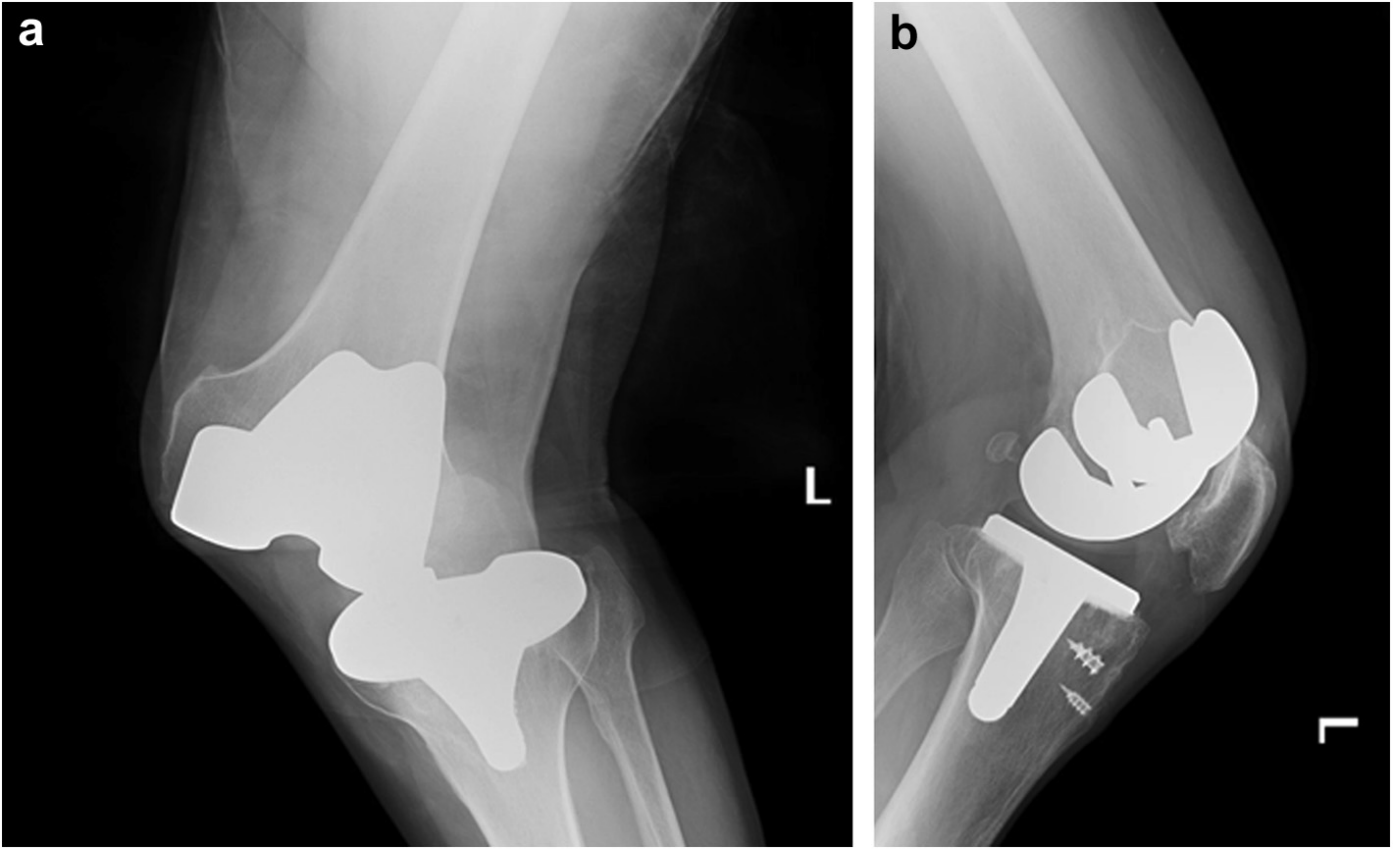
Anteroposterior (a) and lateral (b) radiographs demonstrating a left lateral TKA dislocation. Also noted are Arthrex Corkscrew FT anchors implanted at the time of primary TKA surgery.

**Figure 2:**
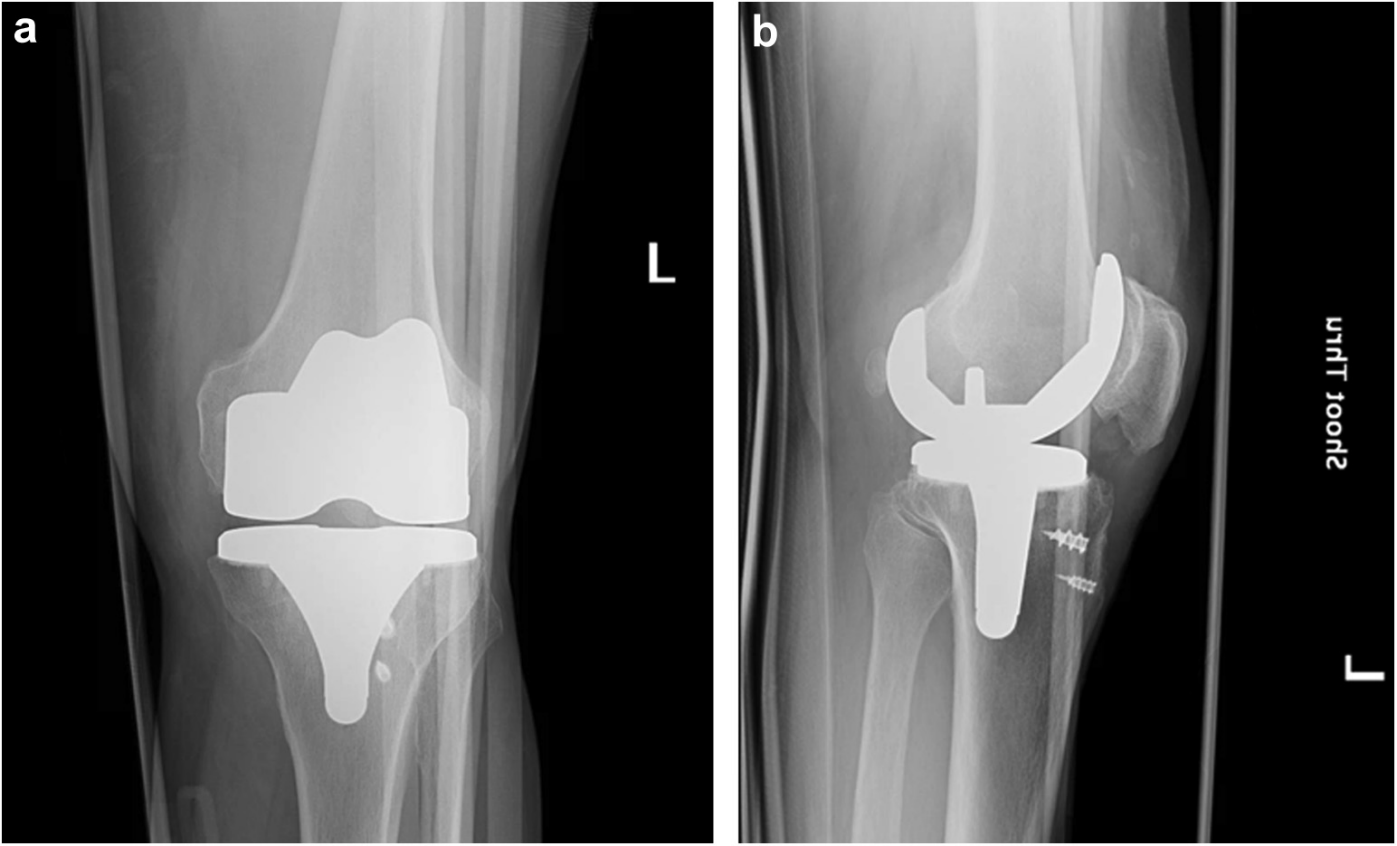
Anteroposterior (a) and lateral (b) radiographs demonstrating interval reduction of a left lateral TKA dislocation.

After his recovery, the patient was able to resume all prior activities. The patient is an avid cyclist and enjoys frequent long-distance bicycle rides. Six years after his bilateral TKAs, the patient was riding his bicycle when he unintentionally turned his wheel in a patch of mud on the road causing him to fall. He developed severe pain in his left knee and was unable to bear weight. The patient was taken to a nearby medical center where he was found to have a left lateral TKA dislocation (Fig. 1). He underwent a closed reduction and was placed into a knee immobilizer (Fig. 2). The patient was admitted for 24 hours of observation and underwent a computed tomography angiogram of the left lower extremity. Advanced imaging and serial neurovascular examinations demonstrated no loss of motor function, sensation, or vascularity to the limb.

The patient was released from the hospital and presented to the senior author’s office 5 days after the injury as a new patient. His body mass index was 27, and he appeared quite healthy. At that time, he was partial weight-bearing with the use of crutches for ambulation assistance. He had a normal neurovascular examination on initial evaluation, and there was no evidence of a foot drop. The patient’s calf was supple, and his leg compartments were soft. There was no hyperextension deformity in full extension; however, there was significant medial knee laxity with valgus stress. There was some laxity with anterior and posterior drawer testing, but the knee did not sublux or dislocate. At 90° of flexion, there was excessive posteromedial and medial knee laxity with the application of internal rotation of the tibia. A diagnosis of MCL deficiency/rupture was made.

The patient was informed of his treatment options, including risks and benefits of operative and nonoperative management. Operative options included repair or reconstruction of the MCL and revision surgery with conversion to a nonhinged constrained prosthesis. He was also offered nonsurgical treatment with cast immobilization followed by a hinged knee brace (HKB) with the understanding that this regimen may fail and could result in requiring a future surgical procedure to address persistent instability. The patient ultimately chose to undergo conservative management. Because of his significant knee swelling, he was asked to continue the use of a knee immobilizer and return in 1 week for repeat examination and placement into a cylinder cast. The patient returned for his follow-up, and repeat physical examination revealed persistent MCL laxity in extension and at 90° of flexion. He was placed into a long leg cylinder cast and was made weight-bearing as tolerated.

He returned to the office 6 weeks after cylinder cast immobilization. Repeat physical examination at this time revealed no medial knee laxity at 0° or at 90° of flexion. He was then placed into a HKB with progressive knee flexion advancement. The treatment regimen consisted of weight-bearing as tolerated in a HKB from 0°-30° of flexion for weeks 1-3 followed by a weekly 10° increase in knee flexion allowance until he reached 0°-90° at week 9.

**Figure 3:**
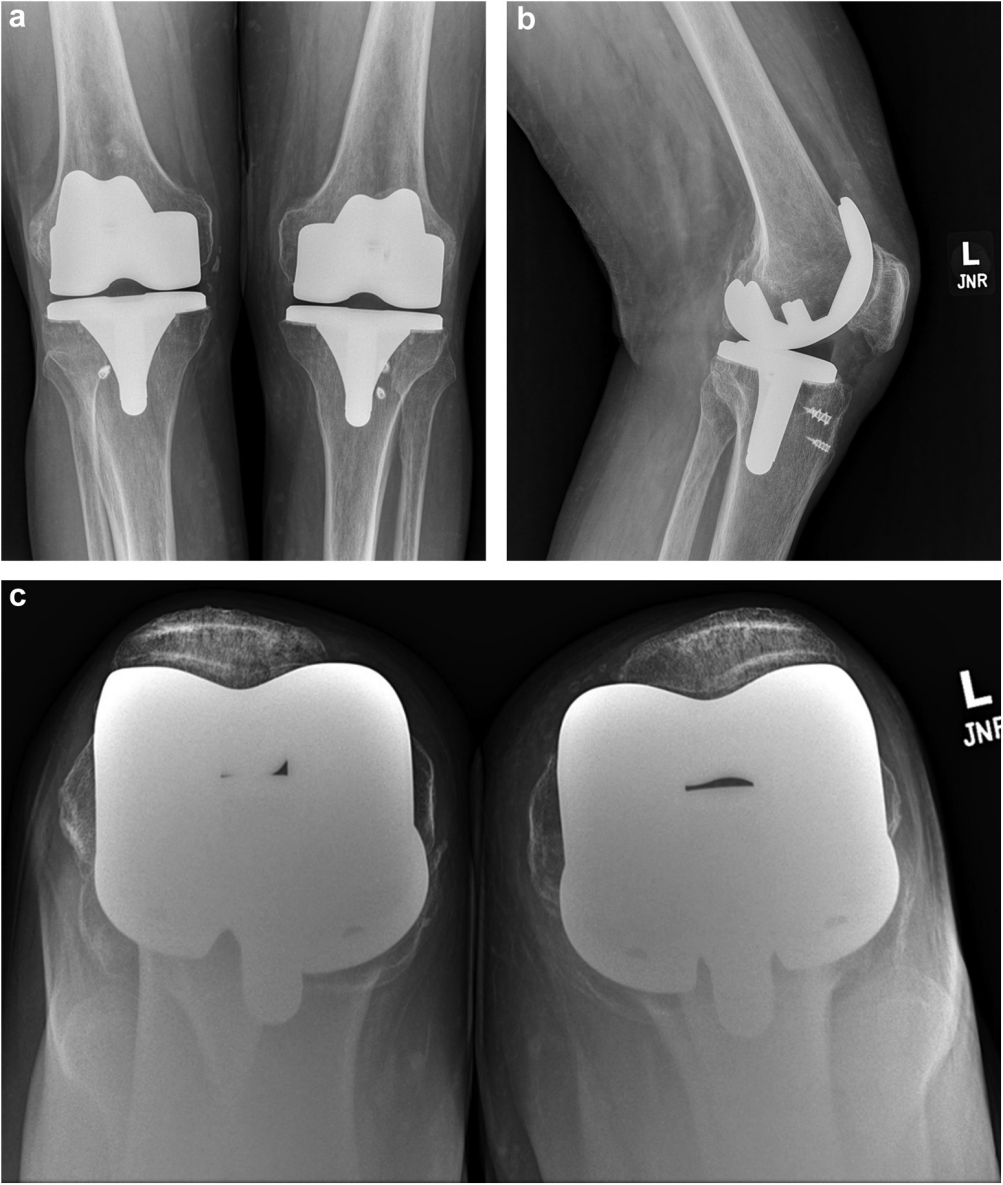
Anteroposterior (a), lateral (b), and sunrise (c) radiographs demonstrating a well-aligned left TKA after successful completion of a conservative treatment regimen for lateral tibiofemoral dislocation with MCL disruption.

The patient was compliant with this treatment regimen and returned to clinic after its completion with full knee range of motion. He was pain free, and there was no medial knee laxity in extension or at 90° of flexion. He was ambulating without the use of an assistive device and was satisfied with the results of his knee function. Radiographs at that time revealed a well-aligned TKA with no evidence of medial joint space gapping (Fig. 3). The HKB was discontinued and his activities were limited to walking and machine exercises in the gym.

Nine months after his injury, the patient reported that he was back to playing doubles tennis and cycling and that his knee felt as stable as it did before the injury. He also stated that his knee was pain free.

## Discussion

Total knee replacement is a life-changing surgery. In most cases, this procedure provides patients an opportunity to resume the level of activity they were used to before the onset of knee arthritis. Some well-known and established limitations of TKA are the ability to kneel, tandem stepping when going up and down stairs, and lateral knee numbness or dysesthesias. A great majority of the time, patients who undergo TKA are highly satisfied with the results and are able to perform activities such as walking, traveling, shopping, driving a vehicle, standing up from a seated position, and light activity.

Some patients, however, have higher performance expectations after TKA. These highly motivated patients want to be able to exercise and play sports, which may even place them at higher average activity levels than nonarthroplasty patients in a similar age group. Suffice to say, some activities seen in patients who have undergone TKA include running, jumping, playing tennis, golf, pickle ball, bicycling, swimming, scuba diving, racquetball, going up and down ladders, driving an 18-wheeler truck, snow skiing, and more.

When patients who have had a prior TKA perform these activities, which are higher demand than what is routinely observed in their age group, they may experience unique injury patterns to the replaced knee joint. This may result in implant loosening, polyethylene wear, periprosthetic fracture, patellar fracture, extensor mechanism disruption, and dislocation of the tibiofemoral or patellofemoral joints. Many times, these events can be catastrophic and may result in revision surgery. Further surgery on the replaced joint can result in infection, failure, and even amputation in extreme cases.

There are many different types of primary total knee implants currently on the market. These include posterior stabilized (PS), CR, highly congruent anterior-stabilized, bicruciate retaining, fixed bearing, mobile bearing, and even gender-specific designs. Each type of implant has its own unique advantages and disadvantages. For example, PS implants have been advocated in patients with patellectomy, inflammatory arthritis, PCL deficiency, and increased body mass index [14,15]. In addition, PS TKA designs make it easier to balance the knee intraoperatively and to achieve femoral roll back. However, owing to the tibial post mechanism, PS TKA designs can be subject to problems such as patellar clunk syndrome, tibial post wear and/or fracture, and, most commonly, patients may complain of a clicking sound in the knee.

CR TKA designs retain the PCL, which should allow for more “natural” kinematics. However, this requires more careful attention to ligament balancing and bone cuts intraoperatively to be able to achieve flexion stability of the implant as well as femoral rollback. One major disadvantage of CR TKA designs is PCL attenuation over time. If the PCL ruptures, then the flexion gap can become unstable, resulting in flexion instability [14,15]. The highly congruent or UC TKA designs are essentially a CR femur which uses a highly congruent tibial insert without the tibial post. The surgical technique generally involves removal of both the anterior cruciate ligament and PCL. Therefore, flexion and extension gaps must be balanced intraoperatively with the use of the UC bearing; however, the advantage is that there is no tibial post wear, tibial post breakage, or clicking with the UC designs [16]. Unlike the CR TKA components, there is a low risk of developing flexion instability because the PCL has already been removed, and the polyethylene insert is designed to achieve increased congruency with the femoral component, further stabilizing the joint in dynamic flexion.

In this study, the original surgeon implanted a TKA design which consisted of a highly congruent anterior-stabilized tibial bearing. It is possible that this bearing selection may have contributed to the success of nonoperative management in this patient comparted to a CR design with retention of the PCL. If the patient had a CR implant instead, then the knee dislocation that he suffered may have also ruptured the PCL and lead to the development of flexion instability despite MCL healing with cast immobilization alone.

## Summary

This is the first case report of a successfully treated traumatic lateral TKA dislocation with associated MCL rupture without surgery. The patient discussed in this case report was treated with cylinder cast immobilization for 6 weeks, followed by hinge knee bracing for an additional 9 weeks. After his conservative treatment, he was gradually able to resume his activities to the same degree of performance he had before the traumatic rupture of his MCL. In the sports medicine literature, grade III MCL ruptures are usually treated with immobilization for 4 to 8 weeks. This is a well-established treatment protocol unless there are other associated cruciate ruptures or meniscal tears. In those cases, surgery may be indicated. However, in the case of MCL rupture in the setting TKA, this case report demonstrates that orthopedic surgeons can successfully treat these patients conservatively.

## Conflicts of interest

C.S. was an unpaid consultant for Biomet and Kyocera (formerly Renovis), has stock or stock options in Pacira and is an AAHKS Abstract Reviewer, Publications Committee member ofAAHKS, and reviewer for the Journal of Arthroplasty Today.

## Informed patient consent

The authors declare that informed patient consent was taken from all the patients.

## Supplementary data

Conflict of Interest Statement for Dubose

Conflict of Interest Statement for Shahrdar

Conflict of Interest Statement for Smidt
