# Supplementary figures and images for: Traumatic Lateral Knee Dislocation of a Well-Functioning Total Knee Arthroplasty: A Case of Medial Collateral Ligament Rupture
Source: Arthroplast Today. 2021 Sep 30;11:168–72. doi: 10.1016/j.artd.2021.08.012 (PMC8495707; doi:10.1016/j.artd.2021.08.012)

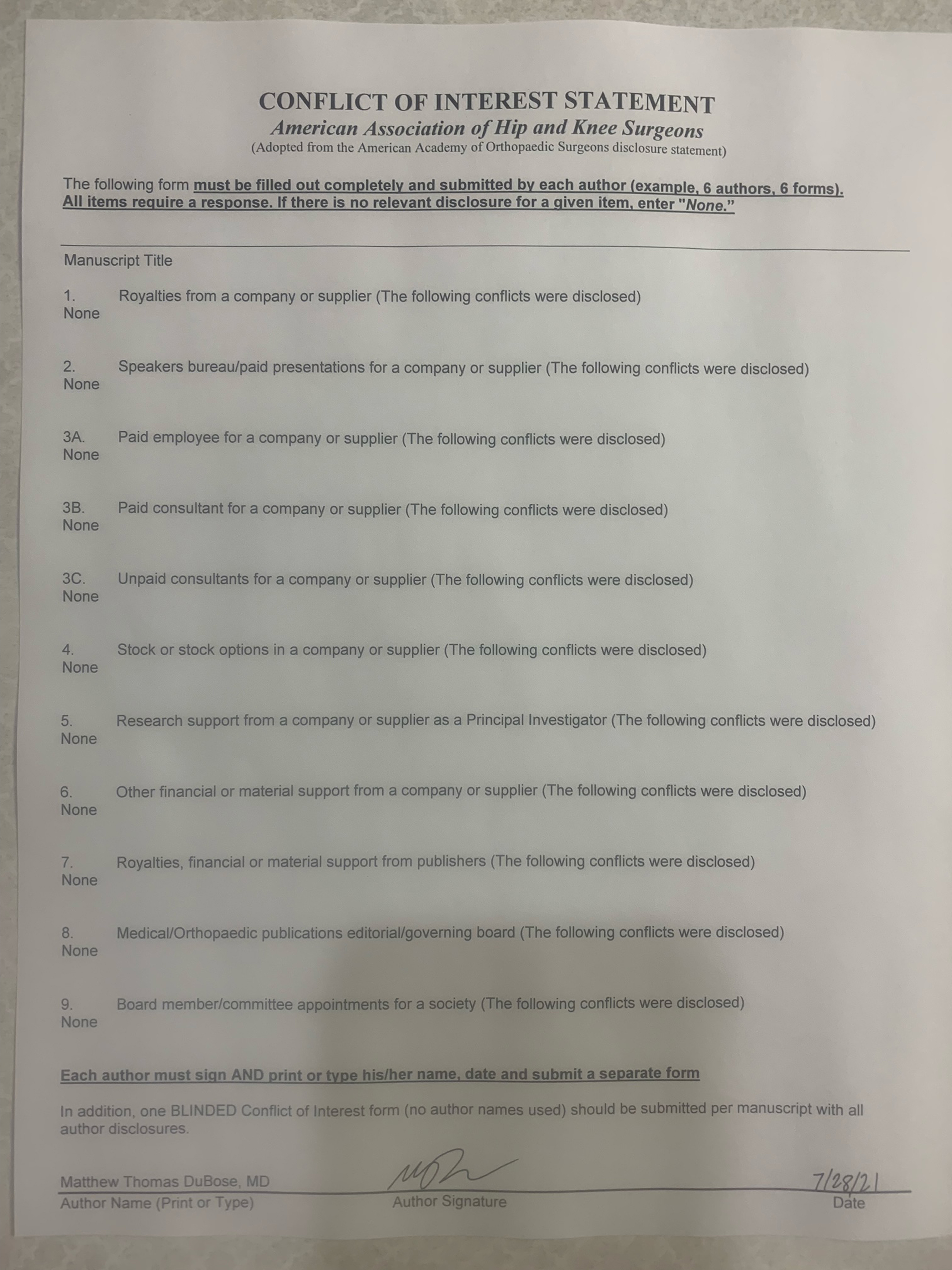

Supplement: Conflict of Interest Statement for Dubose [file figs1.jpg]
