# Supplementary material for: Traumatic Lateral Knee Dislocation of a Well-Functioning Total Knee Arthroplasty: A Case of Medial Collateral Ligament Rupture
Source: Arthroplast Today. 2021 Sep 30;11:168–72. doi: 10.1016/j.artd.2021.08.012 (PMC8495707; doi:10.1016/j.artd.2021.08.012)
Supplement: Conflict of Interest Statement for Shahrdar [file mmc1.docx]

# CONFLICT OF INTEREST STATEMENT

***American Association of Hip and Knee Surgeons***

(Adopted from the American Academy of Orthopaedic Surgeons disclosure statement)

The following form **must be filled out completely and submitted by each author (example, 6 authors, 6 forms).**

**All items require a response. If there is no relevant disclosure for a given item, enter "*None*.”**

Traumatic Lateral Knee Dislocation of a Well-Functioning Total Knee Arthroplasty: A Case of Medial Collateral Ligament Rupture

Manuscript Title

1. Royalties from a company or supplier (The following conflicts were disclosed) **NONE**

2. Speakers bureau/paid presentations for a company or supplier (The following conflicts were disclosed) **NONE**

3A. Paid employee for a company or supplier (The following conflicts were disclosed) **NONE**

3B. Paid consultant for a company or supplier (The following conflicts were disclosed) **NONE**

3C. Unpaid consultants for a company or supplier (The following conflicts were disclosed)

Biomet in the past

Kyocera (formerly Renovis) in the past

4. Stock or stock options in a company or supplier (The following conflicts were disclosed)

Pacira in the past

5. Research support from a company or supplier as a Principal Investigator (The following conflicts were disclosed)

**NONE**

6. Other financial or material support from a company or supplier (The following conflicts were disclosed)

**NONE**

7. Royalties, financial or material support from publishers (The following conflicts were disclosed)

**NONE**

8. Medical/Orthopaedic publications editorial/governing board (The following conflicts were disclosed)

AAHKS Abstract Reviewer

Publications Committee member for AAHKS

Reviewer for the Journal of Arthroplasty Today

9. Board member/committee appointments for a society (The following conflicts were disclosed) **NONE**

**Each author must sign AND print or type his/her name, date and submit a separate form**

In addition, one BLINDED Conflict of Interest form (no author names used) should be submitted per manuscript with all author disclosures.

Cambize Shahrdar MD
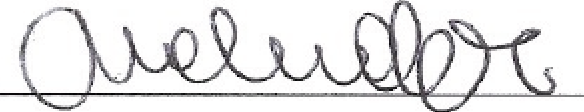
 July 27, 2021

Author Name (Print or Type) Author Signature Date
